# Infants look longer at colours that adults like when colours are highly saturated

**DOI:** 10.3758/s13423-019-01688-5

**Published:** 2019-12-17

**Authors:** A. E. Skelton, A. Franklin

**Affiliations:** Sussex Colour Group, University of Sussex, Brighton, UK

## Abstract

The extent to which aesthetic preferences are ‘innate’ has been highly debated (Reber, Schwarz, & Winkielman, *Personality and Social Psychology Review, 8*(4), 364–382, 2004). For some types of visual stimuli infants look longer at those that adults prefer. It is unclear whether this is also the case for colour. A lack of relationship in prior studies between how long infants look at different colours and how much adults like those colours might be accounted for by stimulus limitations. For example, stimuli may have been too desaturated for infant vision. In the current study, using saturated colours more suitable for infants, we aim to quantify the relationship between infant looking and adult preference for colour. We take infant looking times at multiple hues from a study of infant colour categorization (Skelton, Catchpole, Abbott, Bosten, & Franklin, *Proceedings of the National Academy of Sciences of the United States of America, 114*(21), 5545–5550, 2017) and then measure adult preferences and compare these to infant looking. When colours are highly saturated, infants look longer at colours that adults prefer. Both infant looking time and adult preference are greatest for blue hues and are least for green-yellow. Infant looking and adult preference can be partly summarized by activation of the blue-yellow dimension in the early encoding of human colour vision. These findings suggest that colour preference is at least partially rooted in the sensory mechanisms of colour vision, and more broadly that aesthetic judgements may in part be due to underlying sensory biases.

**Electronic supplementary material:**

The online version of this article (10.3758/s13423-019-01688-5) contains supplementary material, which is available to authorized users.

## Introduction

It is well established that humans have preferences for some visual stimuli over others, for example, preferences for specific faces, patterns, colours or spatial compositions (Fancher, 1996; Palmer & Schloss, 2010; Rhodes, Hickford, & Jeffery, 2000). The source of these perceptual and aesthetic preferences is debated (Ramachandran & Hirstein, 1999; Reber, Schwarz, & Winkielman, 2004). One idea is that preferred visual stimuli have characteristics that are optimal for the human visual system to process, and therefore that these preferences are to some extent ‘innate’ (Krentz & Earl, 2013; Ramachandran & Hirstein, 1999). In support of this idea, there is evidence that infants look longer at visual stimuli that adults prefer. For example, infants look longer at attractive (as judged by adults), than unattractive faces (human, Damon, Mottier, Méary, & Pascalis, 2017; nonhuman, Quinn, Kelly, Lee, Pascalis, & Slater, 2008), and longer at patterns with vertical than horizontal symmetry or asymmetrical patterns which are less preferred by adults (Bornstein, Ferdinandsen, & Gross, 1981). Infants also look longer at original art that adults prefer than art where the balance or focus has been altered (Krentz & Earl, 2013). However, in the case of colour preferences, the relationship between infant looking and adult preference is less clear.

Colour preference might seem a personal and idiosyncratic phenomenon. However, studies have repeatedly found a consistent pattern of colour preference in adults: On average, preference ratings follow a smooth curve as colours vary in hue, with a preference maximum at blue, a minimum at dark yellow, and with cool colours generally preferred over warm colours (Hurlbert & Ling, 2007; Palmer & Schloss, 2010). This pattern of colour preference has been measured as far back as 1897 (Jastrow, 1897), and the broad pattern of preference is generally consistent across industrialized cultures (see Taylor, Clifford, & Franklin, 2013, for an example of cultural variation). Although there is evidence that colour preference varies because of experiential factors (Schloss, Poggesi, & Palmer, 2011), there are also various theories of colour preference which have argued that patterns of colour preference are in some way ‘natural and universal’ and that at least some aspect of colour preference is ‘innate’. For example, ecological valence theory (EVT; Palmer & Schloss, 2010) proposes that people like/dislike colours to the extent that they like/dislike the objects that are associated with the colours. Whilst such associations can be learnt during an individual’s lifetime (as demonstrated by colour preference changing after experience with liked or disliked objects of that colour (Strauss, Schloss, & Palmer, 2013), EVT also suggests that innate colour preferences could draw humans to entities which are evolutionary beneficial (e.g., clean water) and away from those which are not (e.g., rotting waste). Others (e.g., Hurlbert & Ling, 2007) have highlighted how patterns of colour preference can be effectively summarized in terms of the two fundamental neural dimensions that underlie early colour encoding (the ‘red-green’ and ‘blue-yellow’ cone-opponent processes), perhaps suggesting that these colour preferences are rooted in the basic mechanics of the visual system rather than higher level conceptual thought.

There have been a range of studies on infant colour preference. For example, one previous study with a stimuli set designed to test whether infants prefer primary to secondary colours did not find a systematic effect (Franklin et al., 2008). Further weight to claims of the ‘innateness’ of colour preference would be given if a relationship between infant looking and adult colour preference was established. Several studies have found that infants look longest at blue hues and least at yellow or yellow-green (Adams, 1987; Bornstein, 1975; Franklin, Bevis, Ling, & Hurlbert, 2010; Franklin et al., 2008; Teller, Civan, & Bronson-Castain, 2004; Zemach, Chang, & Teller, 2007), these maxima and minima in looking times do broadly correspond respectively to adults’ highly liked and disliked colours (Hurlbert & Ling, 2017; Taylor, Schloss, Palmer, & Franklin, 2013). Other studies have found a less favourable correspondence. In one study, infants were found to look longer at yellow than blue (Adams, 1987). These discrepancies between infant and adult studies could be the differences in lightness and saturation of stimuli across studies, as hue preferences interact with lightness and saturation (Camgöz, Yener, & Güvenç, 2002; Palmer & Schloss, 2010). Only two studies have tested infants and adults using the same stimulus set. One study tested eight colours that were radiant and monochromatic lights (untypical of natural surfaces) and found that infants generally looked longer the more adults liked a colour (Bornstein, 1975). Another study tested only four hues at two lightness and saturation levels, and found no relationship between infant looking time and adult preference (e.g., infants did not look longer than chance at blue hues; Taylor et al., 2013). However, stimuli were relatively desaturated, and it is possible that the discrepancy between infants and adults was due to infants’ poor discrimination of blue-yellow differences (tritan colour vision) at low saturation (e.g., see Teller, Brooks, & Palmer, 1997), and that a stronger relationship between infant and adult measures would exist at higher saturations.

In the current study, we aim to establish whether infants do look longer at colours the more adults like them by using a more comprehensive and suitable stimulus set than prior studies. We reanalyze data from a study of infant colour categorization (Skelton, Catchpole, Abbott, Bosten, & Franklin, 2017) to obtain infant looking times for various hues, and measure adult preferences for these same hues. Skelton et al.’s infant study investigated colour categorization (not preference) using a novelty preference method where 4–6-month-old infants were first familiarized to one hue and then shown a novel hue, and separate groups of infants were tested on 13 different hue pairs. In order to identify infants’ pattern of colour preference, we analyze the length of time infants spent looking on the first trial of the familiarization phase, giving looking times for 14 hues. We analyze the first trial of familiarization rather than the total looking time across all eight familiarization trials since differences between stimuli are likely to wash out over time as infants habituate to stimuli (as is required for the novelty preference method). Hues were reflective stimuli that are more typical of surfaces in the natural world than computer rendered or light-based stimuli, were at a constant lightness level and were at or close to the highest saturation levels for each given hue. Based on prior studies of infant colour vision (Knoblauch, Vital-Durand, & Barbur, 2001) , it is anticipated that 4–6-month-old infants would be able to easily detect all stimuli, and that both red-green and blue-yellow discriminations could be made at such high saturation levels. We relate infant looking times to these hues to adult ratings of colour preference. Although there are many factors which undoubtedly have an effect on adult colour preference, here we investigate how a common factor to both infants and adults, the mechanisms underlying colour vision, can influence response to colour. This is achieved by relating infant looking times and adult colour preference to how the hues activate the ‘red-green’ and ‘blue-yellow’ dimensions of colour encoding (as done for adults in Hurlbert & Ling, 2007, and infants in Franklin et al., 2010), to identify the extent to which infant looking time and adult preference can be summarized by basic sensory processes that underpin colour vision.

## Method

### Participants

#### Infants

Data from 295 4–6-month-old infants who took part in a study on colour categorization in infants (Skelton et al., 2017) was analyzed. The final data set consisted of 201 infants (97 male, *M*_age_ = 21.23 weeks, *SD* = 2.47), as 94 infants were excluded for the following reasons: fussiness (*n* = 78), lack of looking during the critical first trial (*n* = 7), family history of colour deficiency (*n* = 3), prematurity (*n* = 3), and experimenter or equipment error (*n* = 3). All infants were full term, weighed over 2,500 g at birth, and had no known neurological or visual conditions, and parents reported no family history of colour deficiency.

#### Adults

Forty adults (5 males) from the University of Sussex (*M*_age_= 20.63 years, *SD* = 3.21) took part. All participants were screened for colour vision deficiency using Ishihara’s test for colour deficiency (Ishihara, 1917).

### Stimuli and setup

As we are interested in the relationship between infants, adults, and cone contrast methods, it was critical that the same stimuli set is used for both adult and infant participants. The colours were sampled from the World Colour Survey stimulus array, a set of 320 colours in the Munsell system which vary in Munsell value (lightness), hue, and are at high chroma (similar to colourfulness, or saturation) for what is possible for each stimulus. Stimuli were 14 hues sampled at regular intervals of Munsell hue around the colour circle at one lightness level (Munsell value 4, luminance Y = 12cd/m^2^). All chromatic differences between hue and background were larger than the average chromatic threshold at 4-mo (reported to be 21 ΔE in CIELAB colour space; Knoblauch et al., 2001: the smallest difference was 63 ΔE, and the average was 92.89 ΔE). Equating chroma and lightness for all stimuli would likely result in stimuli much closer to or even below these discrimination thresholds reported previously for infants, meaning we’d be unable to test the hypothesis that infant and adult colour preferences relate at higher chroma levels.

**Fig. 1:**
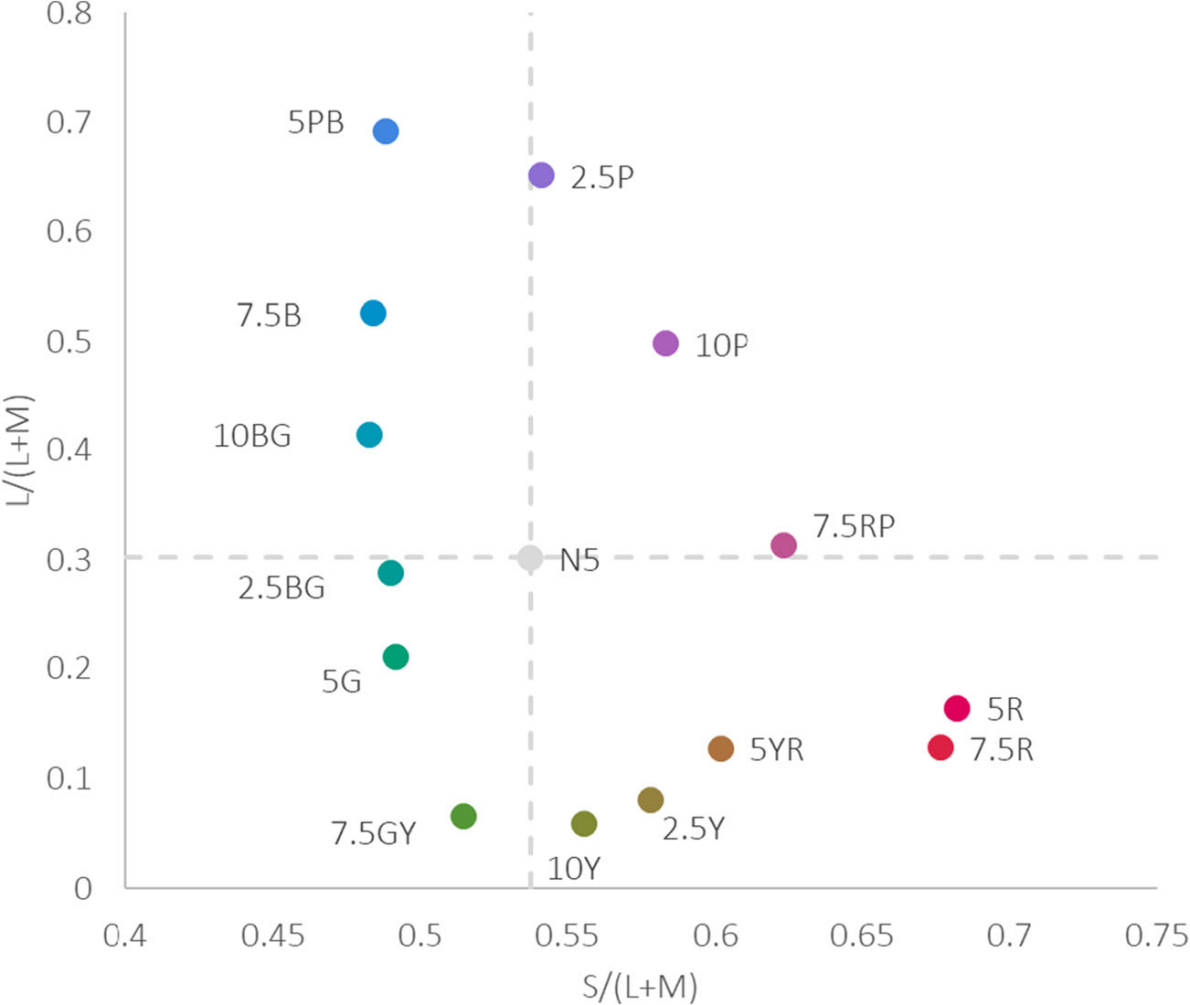
Stimuli plotted in MacLeod-Boynton cone-opponent space with (L/(L+M) and (S/L+M) cardinal axes of colour vision which correspond to activation of the retinogeniculate pathways. The dashed vertical and horizontal lines indicate the background (Munsell N5) to which infants and adults were adapted. The Munsell hue codes for stimuli are given

In the Munsell system hues are divided around the hue circle into hue sectors: red, yellow-red, yellow, green-yellow, green, blue-green, blue, blue-purple, purple, and red-purple, using initials (R, YR, etc.) for simplicity. The Munsell notation of the stimuli is given in the form hue value/chroma. The full notations of stimuli are; 5 R 4/14, 7.5 R 4/14, 5 YR 4/8, 2.5 Y 4/6, 10 Y 4/6, 7.5 GY 4/8, 5 G 4/10 2.5 BG 4/8 10 BG, 4/8, 7.5 B 4/8, 5 BP 4/10, 2.5 P 4/10, 10 P 4/10, 7.5 RP 4/10. We identify stimuli Y as yellow here because of Munsell hue notation, although we recognize that at the Munsell value sampled, these hues are darker than prototypical yellow. Stimuli are also plotted here in a version of the MacLeod Boynton chromaticity diagram (see Fig. 1), where the two axes represent activation in the retinogeniculate pathways that underlie colour vision L/(L+M) (‘red-green’) and S/(L+M)) (‘blue yellow’). The conversion of stimuli from CIE xyY to the MacLeod Boynton Chromaticity Diagram was carried out with reflectance spectra taken from the University of Joensuu Colour Group database (https://wwww.ue.fi/en/web/spectral/spectraldatabase), the Stockman and Sharpe 2° cone fundamentals (Stockman & Sharpe, 2000), and a D65 illuminant (which matches the illumination the colours were viewed under).

For the infants, colours were presented as two 12cm squares in a viewing booth painted with Munsell N5 paint (luminance Y = 19.77 cd/m^2^, *x* = 0.312, *y* = 0.325). The stimuli were presented in two windows whose inner edge was 3.5 cm to the left and right of central fixation. Looking preferences for stimuli presented as a pair are equivalent to preferences when stimuli are presented individually (Bornstein, 1975). Side bias to a particular window cannot account for any variation across hue, as hues appear in both the left and right windows at the same time. Infants were seated in a car seat 50 cm away from the centre point of these windows. The visual angle of the stimuli windows meant that infants could quickly saccade between the two windows. Colours were illuminated by an overhead illuminant and by two spotlights angled onto the stimuli from behind the infant to ensure uniform illumination. All lighting was an artificial simulation of natural daylight illumination (D65, 6500k).

Adults’ stimulus colours were identical to those used in the infant experiment. Each colour was presented as a 5-cm square mounted on card painted with Munsell N5 paint. A table was set up in the infant testing booth allowing adult participants to view the colours under the same illumination condition as the infants.

### Design and procedure

Infant data are taken from the looking times in Skelton et al.’s (2017) colour categorization study. Here, for the purpose of examining colour preference, the looking time from the first 8-second trial in Skelton et al. is analyzed. In Skelton et al., prior to the first trial, infants were centrally fixated with a black and white looming attention-getter displayed on a small screen between the two windows of the booth. Once the attention-getter was centrally fixated, two identical squares of colour were lowered into the viewing windows, and an experimenter, blind to the condition, coded infant looking to the colours using a MATLAB program whilst viewing the infant via a webcam. Each infant saw one hue, and there was a minimum number of 10 infants per hue, with infants randomly allocated to each hue. Skelton et al. followed an optional stopping procedure that is routine when using Bayesian analysis. This means that the sensitivity of the Bayes Factors in the analysis of Skelton et al. determined how many infants were tested: Testing stopped when a sensitive Bayes factor was reached in support of either the null or alternative hypotheses (e.g., Rouder, 2014). As a result of this the number of participants allocated to each hue are not equal across hues (average *N* per pair = 14.35, *SD* = 4.52).

Adults were shown colours individually in a random order twice and were asked to rate their preference for the colours on a scale ranging from 0 to 100 by making a mark on the line that represented the scale.

## Results

As in Skelton et al. (2017), traditional null hypothesis significance testing is accompanied by Bayes factors to aid with the interpretation of the data. Bayes factors allow richer interpretation of the strength of the evidence for either the null or alternative hypothesis (Dienes, 2014), and in the case of regression they allow us to assess the predictive strength of each possible regression model, without having to correct for multiple comparisons, a process which can increase the chance of Type II errors (Dienes, 2016). A BF of 0.33 indicates evidence for the null hypothesis, and a BF of 3 and above indicates substantial support for the alternative hypothesis. In addition, we used the Robust Correlation Toolbox (Pernet, Wilcox, & Rousselet, 2013) to examine the relationship between variables. Skipped correlations are a more robust measure of correlations involving outlier detection, weighting, and/or removal. If the reported bootstrap confidence intervals include zero, then the null hypothesis cannot be rejected (Rousselet & Pernet, 2012). Taken together, these analyses allow for a reliable insight into the strength of the evidence for the claims reported.

### Comparison of infant looking time and adult preference ratings

**Fig. 2:**
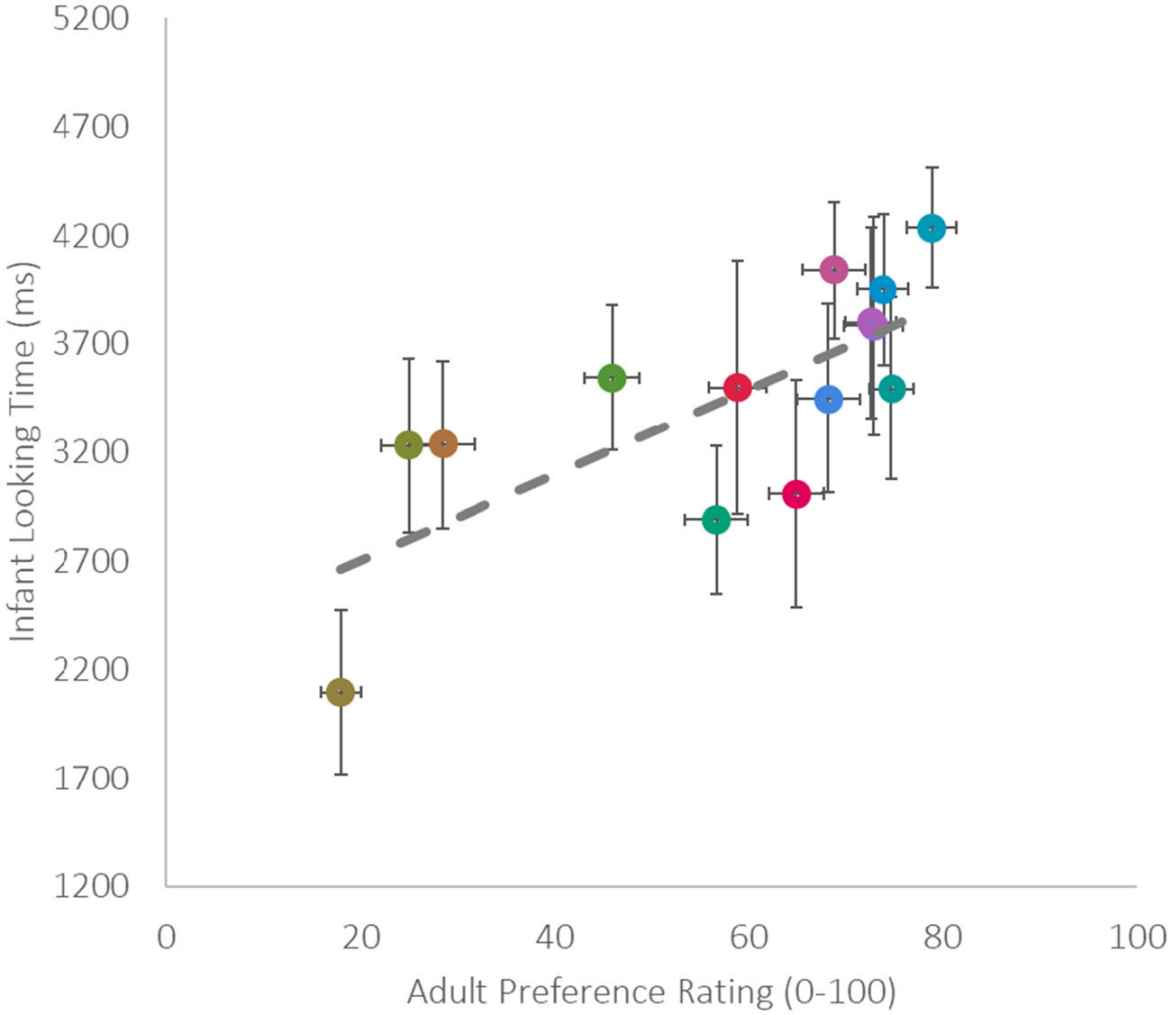
Correlations between average infant looking time (ms) and average adult preference rating (0–100). Data screened with Robust Correlation toolbox for outliers before analysis carried out. Error bars +/- 1 *SE*

Total infant looking times to both left and right squares of colour were averaged across infants for each hue. Infants looked for longest at one of the blue hues (10 BG, *M* = 4,236 ms, *SD =* 1,180.31), twice as long as the hue they looked at least, which was one of the yellow hues (2.5 Y, *M* = 1,996 ms, *SD =* 1,239.60). Adults maximum and minimum preference ratings were for the same hues that infants looked longest and least at, with the highest preference for 10 BG (*M* = 78.86, *SE =* 2.52), and lowest for 2.5 Y (*M* = 18.02, *SE =* 2.11; see Fig. 2), the overall pattern of adult preference replicates those found previously. As can be seen in Fig. 2, infants tend to look longer the more adults prefer the colour. This was confirmed with by a significant robust correlation (Pernet et al., 2013) between infant looking time and adult preference ratings across hues, skipped correlation, *r* = 0.73, *t = 3*.71, CI [0.40, 0.91], BF 13.97.^1^ There was no significant correlation between adult and infant measures and chroma (skipped Spearman correlation infant: *r* = .16, CI [−0.45, 0.68], BF 0.78; adult: *r* = .27 *t* = 0.98, CI [−0.46, 0.79], BF 0.88.

### Sensory mechanisms of colour preference

**Fig. 3:**
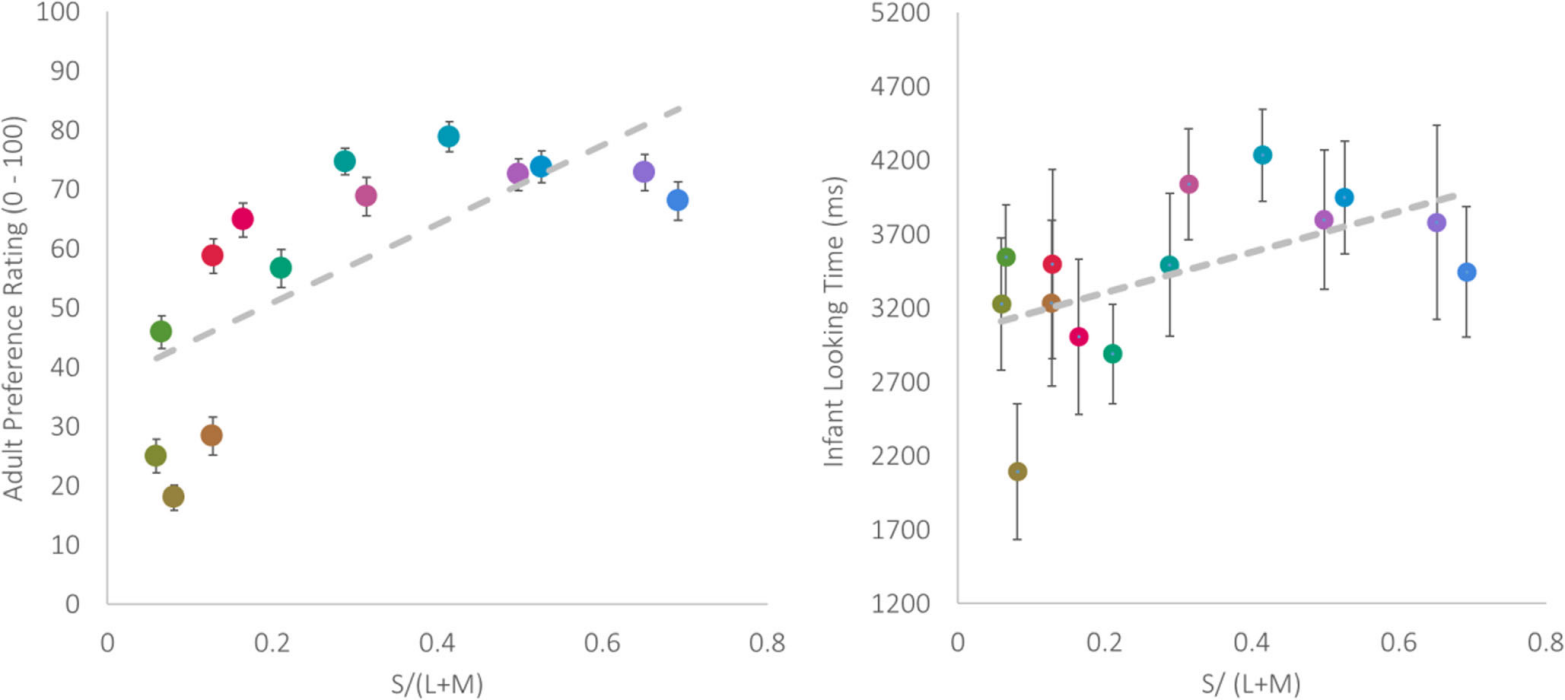
Correlations of average adult preference rating (0–100) and S/(L+M) (left), and average infant looking time (ms) and S/(L+M) (right). Error bars +/- 1 *SE*

In order to assess whether the sensory mechanisms that underpin colour vision could summarize infant looking times or adult colour preference, a series of regression analyses were conducted. To determine which model, L/(L+M) or S/(L+M), or a combination of both, is most accurate at predicting infant and adult response to colour, a stepwise regression with L/(L+M) first, and S/(L+M) second as predictor variables for infant looking time and adult preference. Bayes factors were calculated using an R Package, BayesFactor (Morey, Rouder, Jamil, & Morey, 2015) for all possible models. The stepwise regression found that a significant amount of the variance in infant looking time could be predicted from S/(L+M) alone, with infants looking for longer at bluer colours (greater S/(L+M) activation), *R*^2^= 0.304, *F*(1, 12) = 5.23, *p* = .041, Beta = 0.551 (see Fig. 3). However, the BF for this model was 2.02, suggesting there is not firm evidence in this data set for the experimental hypothesis (BF for both L/(L+M) and L/(L+M)&S/(L+M) models were also insensitive, BF = 0.54, BF *=* 0.97, respectively).

For adults, the stepwise regression also found that the best model was S/(L+M) alone, and adults preferred colours more which were bluer (greater S/(L+M) activation), *R*^2^ = 0.515, *F* = 12.76, *p* = .004, Beta = 0.718, BF = 10.41 (see Fig. 3). Note, the Bayes factor for a model with both L/(L+M) and S/(L+M) was 4.14, demonstrating that a model with both predictors would also predict variation in adult preference ratings, although to a lesser extent than S/(L+M) alone.

## Discussion

The current study aimed to quantify the relationship between how long infants look at colours and how much adults like those colours. We found a striking correspondence between infant looking and adult liking. The colour looked at least by infants was the most disliked by adults and the colour looked at most by infants was the most liked by adults. The relationship between infant and adult measures was significant, and infant looking times accounted for over half of the variance in adult preferences. Infant looking and adult preference were also both best accounted for by a sensory model of colour vision which quantified how the colours activate the ‘blue-yellow’ S/(L+M) retinogeniculate pathway. Variation in chroma of the stimuli set (necessary for all hues to be easily discriminable for infants at a single lightness values) cannot account for these relationships, as chroma does not correlate with either adult or infant response to hues.

Even though infant data are often very noisy, many studies in infant cognition have been shown to replicate across a range of methods and measures. In the current study, the pattern of infant looking across colours, where infants look longest at blue hues and least at yellow/green hues was similar to several prior infant studies (e.g., Bornstein, 1975; Franklin et al., 2010; Franklin et al., 2008; Teller et al., 2004; Zemach et al., 2007; see also Brown & Lindsey, 2013). The current study elaborates on the pattern of infant looking by using a far more extensive stimulus set than in prior studies. The stimulus set in the current study also had colours that were well above infant chromatic thresholds at 4–6 months and which were at high saturation levels to guard against the concern that infants cannot make tritan discriminations at low saturation. Given the correspondence between infant looking and adult liking in the current study when stimuli are at high saturation, we propose that the different pattern of infant looking and lack of infant and adult correspondence in Taylor, Schloss et al. (2013) was due to stimuli being desaturated.

Adult colour preferences, and to a lesser extent infant looking times at colours could be effectively summarized by the underlying sensory mechanisms of colour vision. The fact that S/(L+M) along was the best predictive model of both infant looking and adult preference provides further weight to the argument that infants and adults have a similar pattern of response to the colours. Previous studies, also report that adults give a positive weighting to S/(L+M), with adults rating more blueish hues as more preferred (Hurlbert & Ling, 2007), although one prior study found that infant looking is best summarized by L-M (Franklin et al., 2010). We propose that this discrepancy can again be explained by infants’ poor tritan discrimination at low saturation levels—when stimuli are highly saturated as in the current study and unlike in Franklin et al. (2010), S/(L+M) is the strongest predictor of infants’ response just like for adults. For both adults and infants, the relationship between S/(L+M) and their response does appear to be somewhat curvilinear (with their response peaking at blue not at maximum S/L+M), which explains why a simple linear model of sensory mechanisms only predicts around half of the variance in response. More complex modelling of other colour spaces may capture infant colour preferences better than just cone-opponency, as in adults (Schloss, Lessard, Racey, & Hurlbert, 2018). It is clear from many adult colour preference data that interactions between the hue, saturation, and lightness dimensions of colour can influence colour preference. Nevertheless, the relationship of adult colour preference with S/(L+M) and also with infant looking times provides further weight to the argument that colour preference is at least partially rooted in the sensory mechanisms of our colour vision.

Of course, how long infants look at colours cannot completely account for how much adults like colours—just over half of the variance in adult preference is explained. Adult colour preference has been shown to be related to a number of other measures such as protoypicality or object valence, and the relationship we reveal between infants and adults flags the need for further consideration in how these factors interact. This leaves room for other experiential factors to contribute to adult colour preference such as the associations between colour and objects that are made over a lifetime (e.g., Palmer & Schloss, 2010). We are not suggesting that colour preferences are completely ‘innate’.

We also do not suggest here that how long an infant looks at a colour reflects their affective or aesthetic response to colour. Infants might look longer at stimuli for reasons other than liking those stimuli. Novelty or complexity is known to drive infants’ looking response—for example, infants look for longer at a fearful face than a happy face (Peltola, Leppänen, Palokangas, & Hietanen, 2008). Adults do look longer at stimuli that they like (Taylor, Schloss et al., 2013), yet we are unable to determine from this and prior studies whether this is the case for infants (see Taylor, Schloss et al., 2013). Further research which aims to identify how infants’ sensory biases to some colours over others relates to conscious and explicit affective response to colour across the developmental lifespan is needed. For now, we establish that when colours are sufficiently saturated for infants to see there is a high degree of similarity between how long infants look at those colours and how much they are liked by adults.

## Electronic supplementary material

ESM 1 (XLSX 17 kb)

## Footnotes

1 An anonymous reviewer requested the correlation be conducted without the data point from the 2.5 Y stimulus, due to concern over the lower chroma of this hue having undue influence over the relationship between infant looking time and adult preference. The robust correlations that we conduct do appropriately deal with potential outliers. Nevertheless, when this data point is removed as requested, there remains a significant correlation between infant looking time and adult preference, *r* = .58, *p* = .034, CI [0.02, 0.93], BF = 4.30.
